# Excursion to the Mediterranean

**Published:** 1874-12

**Authors:** 


OUR READERS WILL RECALL the excursion of the steamer "Quaker City," from New York, in the year 1867, to the shores of the Mediterranean, the Holy Land, and to Egypt, which furnished materials for the humorous work of Mark Twain, styled the "Innocents Abroad." It is now proposed to repeat the excursion during the coming season in a first-class ocean steamer, to leave New York about the first of June. The steamship will land at the following points upon the route: Fayal, Cadiz, Gibraltar, Malaya, Marseilles, Genoa, Leghorn, Naples, Palermo, Athens, Constantinople, Sevastapol, Smyrna, Beyrout, Mount Carmel, Joppa, and Alexandria. This is certainly an attractive programme for any of our readers whose leisure and inclination may lead them to embrace it. To such we can heartily commend it as promising rich stores of recreation, instruction and enjoyment. Mark Twain very pointedly remarks: "Travel is fatal to prejudice, bigotry and narrow-mindedness, and many of our people need it sorely on these accounts. Broad, wholesome, charitable views of men and things cannot be acquired by vegetating in one little corner of the earth all one's lifetime." The price of the passage has been fixed at fifteen hundred dollars in greenbacks.

A lady at our elbow suggests the hope that the literateur of the coming excursion will give to the world a less ponderous and more readable account of his travels than did the leader of the band of "innocents."

